# Assessment of Muscle Wasting in Long-Stay ICU Patients Using a New Ultrasound Protocol

**DOI:** 10.3390/nu10121849

**Published:** 2018-12-01

**Authors:** Carmen Rosa Hernández-Socorro, Pedro Saavedra, Juan Carlos López-Fernández, Sergio Ruiz-Santana

**Affiliations:** 1 Department of Radiology, Hospital Universitario de Gran Canaria Dr. Negrín, Universidad de Las Palmas de Gran Canaria, Barranco de la Ballena s/n, 35010 Las Palmas de Gran Canaria, Spain; 2 Department of Mathematics, Universidad de Las Palmas de Gran Canaria, 35010 Las Palmas de Gran Canaria, Spain; p.saavedra.santana@gmail.com; 3 Department of Neurology, Hospital Universitario de Gran Canaria Dr. Negrín, Universidad of Las Palmas de Gran Canaria, Barranco de la Ballena s/n, 35010 Las Palmas de Gran Canaria, Spain; jclopezfdez@gmail.com; 4 Department of Intensive Care, Hospital Universitario de Gran Canaria Dr. Negrín, Universidad of Las Palmas de Gran Canaria, Barranco de la Ballena s/n, 35010 Las Palmas de Gran Canaria, Spain; sruisan@gobiernodecanarias.org

**Keywords:** ultrasound, sarcopenia, quadriceps femoris, muscle weakness, muscle mass, critical illness, Bayesian prediction

## Abstract

There is currently no standardized procedure to assess sarcopenia in long-stay catabolic patients. Our aim is to analyze a novel ultrasound muscle assessment protocol in these patients versus healthy controls, by carrying out a prospective observational study. We designed a new ultrasound protocol that assesses quadriceps rectus femoris (QRF) muscle quality in real-time B-mode, color-Doppler, and M-mode ultrasound, and evaluates QRF intramuscular central tendon thickness, cross-sectional area, and muscle thickness in ultrasound B-mode. Logistic regression was performed as a multivariable analysis on 29 cases and 19 controls. The QRF muscle area and thickness were shown to significantly decrease (*p* ≤ 0.001), and the central tendon thickness significantly increased (*p* = 0.047) in cases versus controls. The QRF muscle echogenicity and angiogenic activity fasciculations, subcutaneous edema, and intramuscular fluid were also significantly different between the two groups (*p* < 0.001). The selected variables in the multivariate logit analysis were the muscle area (OR per cm^2^ = 0.07; 95% confidence interval (CI) = 0.012–0.41) and the central tendon thickness (OR per mm 1.887; 95% CI = 2.66–13.38).

## 1. Introduction

Skeletal muscle wasting is a characteristic early finding in the acute phase response, but has also been linked to functional impairment in patients with prolonged weaning from mechanical ventilation and increased hospital length of stay, as well as acute and long-term functional disability. Nevertheless, its relationship with neuromuscular acquired weakness or secondary sarcopenia has not yet been well-established. Moreover, increased energy and protein administration within the first acute-phase admission week does not seem to prevent muscle wasting or promote muscle preservation. Protein-loading and exercise, although used successfully to treat frailty in older patients [1], may fail in the still not well-defined group of severely catabolic patients, if applied as a standard treatment [2,3].

The most frequently used imaging modalities to measure muscle mass are magnetic resonance (MR), ultrasonography (US), computed tomography (CT), and dual-energy X-ray absorptiometry (DXA). In addition, bioelectrical impedance analysis (BIA) is used to assess body composition [4]. However, muscle strength may be clinically difficult to assess in hospital patients, as many of them are not awake or adequately responsive to evaluation therapies, and thus cannot cooperate with the assessment procedures [3]. In addition to this, muscle thickness wasting is more evident and reaches its peak after the first two to three weeks of an acute-phase catabolic situation [5]. Imaging diagnostic techniques devoted to assessing muscle wasting at hospital admission and throughout the patient’s Intensive Care Unit (ICU) stay, particularly when neuromuscular acquired weakness is suspected, are valuable diagnostic tools. Among the aforementioned techniques, magnetic resonance (MR) and ultrasound (US) have been used to assess skeletal muscle mass in patients with many clinical conditions, displaying an excellent correlation coefficient of 0.99 for muscle measurement [6,7,8,9]. In this regard, low skeletal muscle mass quality at ICU admission, as assessed by computed tomography (CT)-derived density made during the initial days of ICU admission, is an independent risk factor for 6-month mortality in mechanically-ventilated, critically ill patients [7].

In the last few years, many authors have researched and demonstrated the validity of US to assess musculoskeletal quality and quantity status in severely catabolic patients, by studying different muscle groups at different moments during the patients’ ICU stays [10,11]. However, these authors have also performed diverse measurements on different muscles, obtaining disparate results that are difficult to replicate due to insufficient standardization. Therefore, a clear-cut, easy to perform, reproducible, standardized US muscle wasting measurement protocol is clearly needed in clinical practice [11,12,13].

We were already aware of the sonographic importance of studying the quadriceps rectus femoris (QRF), since it is the only anatomically bipennate muscle. It has a unique intramuscular central tendon that extends to the lower third of the muscle between the four quadriceps femoris muscles [14].

The aim of this study was to investigate a new, reliable, structured ultrasonographic protocol to assess muscle quantity and quality sarcopenia in mechanically-ventilated patients with suspected neuromuscular acquired weakness, but without previous malnutrition, who were expected to stay at least seven days in the ICU [15]. To this effect, we ultrasonographically studied the intramuscular central tendon thickness, the muscle cross-sectional area (CSA) and its thickness, and the grade of echogenicity, as well as conducting Doppler and M-mode assessments of vascularization and fasciculations of QRF muscle in these patients. We collected the same data from matched healthy controls.

## 2. Materials and Methods

### 2.1. Patients and Healthy Controls

We conducted a prospective observational study in a 32-bed adult medical–surgical ICU at a tertiary University Hospital in Las Palmas de Gran Canaria (Canary Islands, Spain) between May 2016 and May 2017. As previously mentioned, patients did not have malnutrition prior to their admission, required prolonged mechanical ventilation, and were expected to stay at least seven days in the ICU. In all studied patients, when neuromuscular acquired weakness was clinically suspected [15], the newly designed protocolized QRF muscle US examination was performed. Clinical neuromuscular acquired weakness was considered when the patient, once awake, presented flaccid quadric-paresis and hypo-reflexia in the absence of other neurological, biochemical, or central neurological damage [3], and had a median Medical Research Council (MRC) score of less than four [16,17]. We also conducted identically protocolized US QRF muscle assessments on age, sex, and body mass index (BMI) matched healthy controls. Patients who were not expected to survive longer than three days and those with primary neuromuscular pathology were excluded. The following demographic and clinical data were obtained: age, sex, height, weight, BMI, Glasgow Coma Score (GCS), acute physiology and chronic health evaluation (Apache) II score, sequential organ failure assessment (SOFA) score, ICU admission date, ICU discharge date, admission diagnosis and ICU length of stay (LOS), and presence of sepsis. Additionally, we collected data on the following organic failures on ICU admission: respiratory, cardiovascular, renal, hepatic, hematologic, and gastrointestinal. An ultrasound study was performed the day after ICU admission, as well as a corticosteroid treatment and neuromuscular blocking treatment.

### 2.2. New Quadriceps Rectus Femoris Ultrasonography Assessment Protocol

We performed a thorough US assessment with an Aplio 500 ultrasound system (Toshiba Medical Systems Corporation, Tochigi, Japan) with 10–12 MHz small parts and a multifrequency linear-array probe (width of probe, 38–58 mm) on all patients to whom a neuromuscular acquired weakness diagnosis was considered appropriate, as well as on the healthy controls. During the assessment, the participants lay supine with their arms supinated and knees extended and relaxed to full extension. The probe was coated with an adequate water-soluble transmission gel to provide acoustic contact without depression of the dermal surface, and was aligned perpendicularly to the longitudinal and transversal axes of QRF, with the aim of obtaining transverse and longitudinal images. Several images were registered of both QRF muscles for each measurement site. Image files were stored on the US device computer. Since muscle dimensions change with contraction/relaxation and the studied muscle is more compressible in a relaxed state [18,19,20], the assessment was done without compression. The acquisition site was located two-thirds of the way along the femur length, measured between the upper pole of the patella and the anterior superior iliac spine. We measured the exact site with electronic calipers, so that once the muscle was imaged, its boundaries could be identified and measured. For greater exactitude, averaged measurements were estimated.

**Figure 1:**
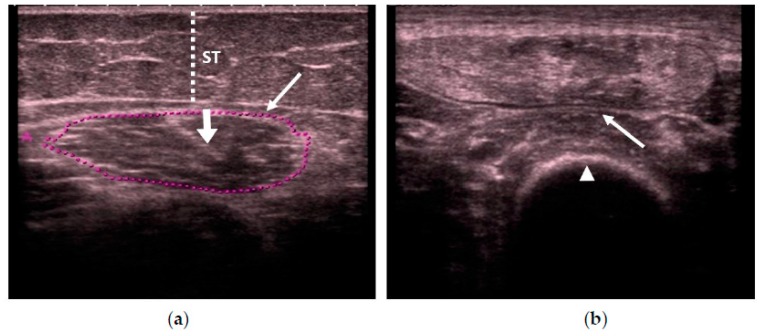

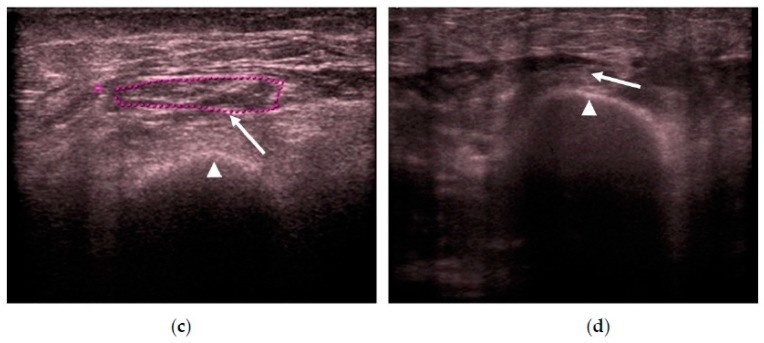
Transversal ultrasonography (US) scan that shows quadriceps rectus femoris (QRF) muscle echogenicity. (**a**) Matched healthy control: Category 1 (normal hypoechogenicity), with the central tendon (thick arrow). (**b**) Category 2 (heterogeneous). (**c**) Category 3 (fat infiltration). (**d**) Category 4 (atrophy due to fasciitis and muscle necrosis). QRF: quadriceps rectus femoris (arrows); femur (arrowhead); ST: subcutaneous cellular tissue (dotted line); US: ultrasound.

In all of the studies, we first used real-time B-mode US scanning to assess the muscle quality according to its echogenicity by using a specifically designed scale, due to the importance of age-related muscle replacement by fat and fibrous tissue or sarcopenia. The scale was as follows (see Figure 1): homogenous hypoechogenicity (Category 1) [18], heterogeneous hypoechogenicity (Category 2) [21], fat infiltration (Category 3) [22,23,24], and with fasciitis and/or necrosis (Category 4) [18,25]. We measured the intramuscular central tendon thickness in mm, with an insonation angle perpendicular to the tendon.

**Figure 2:**
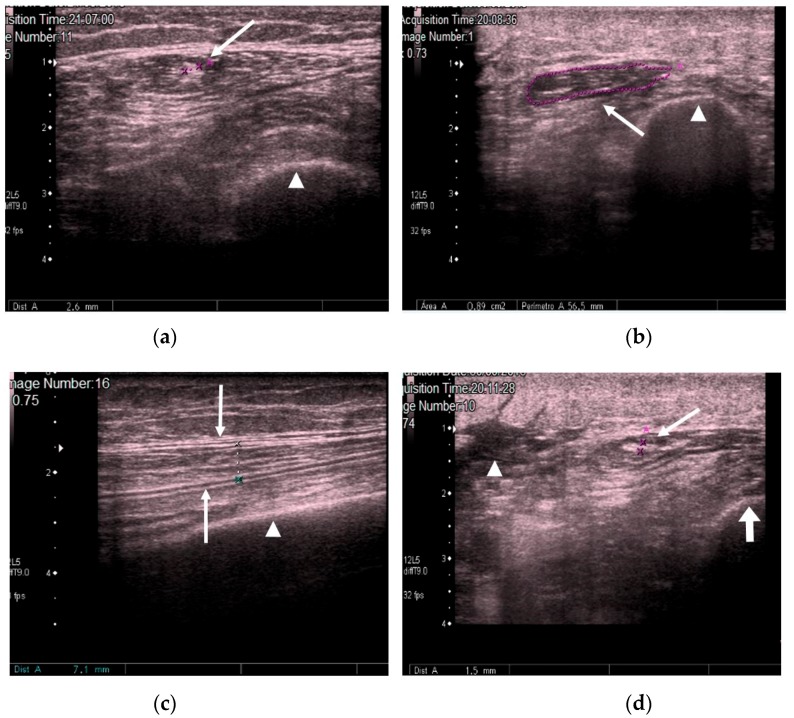
(**a**) Transversal QRF muscle US scan: multiorgan failure, tendon thickness of 2.6 mm (arrow), and femur (arrowhead). (**b**) Transversal QRF muscle US scan: multiorgan failure, cross-sectional area (CSA) of 0.89 cm^2^ (arrow), and femur (arrowhead). (**c**) Longitudinal QRF muscle US scan: muscle thickness of 7.1 mm (arrow) and femur (arrowhead). (**d**) Longitudinal QRF muscle US scan: subcutaneous edema, with intramuscular and interfacial fluid (arrowhead). QRF tendon is 1.5 mm (arrow) with the femur (thick arrow). QRF: quadriceps rectus femoris, US: ultrasound, CSA: cross-sectional area.

We measured the CSA in cm^2^ and muscle thickness in mm, and scanned for the presence or absence of edema in the subcutaneous cellular tissue and the intramuscular and intrafascial fluid (Figure 2).

**Figure 3:**
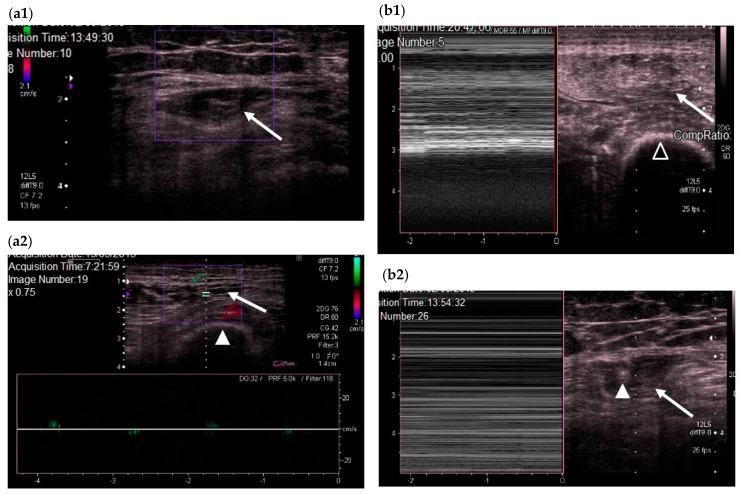
(**a**) Transversal QRF muscle US scan and color Doppler. (**a1**) QRF muscle without vascularization (echogenicity Category 3). (**a2**) QRF muscle with vascularization (echogenicity Category 2): the femur (arrowhead) and the QRF muscle (arrow). (**b**) Transversal US scans and M-mode; (**b1**) QRF muscle (arrow) with fasciculations (echogenicity Category 2) and the femur (black arrowhead); (**b2**) QRF muscle without fasciculations (echogenicity Category 3). QRF tendon of 3.6 mm (white arrowhead) and the QRF muscle (arrow). QRF: quadriceps rectus femoris, US: ultrasound.

We subsequently used color Doppler to evaluate QRF vascularization and M-mode to establish the presence or absence of fasciculations (Figure 3) [18].

The hospital Institutional Review Board approved the study (protocol number: 160049, 27 May 2016). Written informed consent was obtained from patients or close relatives.

### 2.3. Statistical Analysis

Categorical variables are expressed as frequencies, while percentages and continuous variables are represented by the mean and standard deviation (SD) when data follows a normal distribution, or as the median and interquartile range (IQR = 25th–75th percentile) when distribution departed from normality. The percentages were compared appropriately using the Chi-square (χ^2^) test or the exact Fisher test; the means were compared by the *t*-test, and the medians by the Wilcoxon test for independent data. In order to obtain a predictor of the class, a multivariate logistic regression was performed. All discriminating variable potentials for the class were introduced in the analysis, and a selection based on the algorithm of complete enumeration and Bayesian information criterion was carried out. The estimated model was summarized by coefficients (SE), *p*-values, and odds ratios, all of which were estimated by confidence intervals at 95%. A predictive muscle wasting score was obtained from the logistic regression model. For this score, a receiver operating characteristic analysis (ROC) was carried out to determine its discriminant power. The area under the corresponding ROC curve was estimated by means of a 95% confidence interval. The discriminating threshold was chosen from amongst those that minimized the function, closest to the upper left-hand corner:

(1 − sensitivity)^2^ + (1 − specificity)^2^

For the obtained predictor, the sensitivity and specificity were estimated by means of 95% confidence intervals.

Statistical significance was set at *p* < 0.05. Data were analyzed by using the R package, version 3.3.1 (R Development Core Team, 2016).

## 3. Results

During the study period, 862 patients were admitted to the ICU, where 738 of them were mechanically ventilated and 159 had prolonged mechanical ventilation (median (IQR) for number of days: 37.5 (28.8; 61.9)). Neuromuscular acquired weakness was clinically suspected in 29 patients to whom the QRF-US protocol was applied. Time on mechanical ventilation was 27 days (IQR: 15.5; 48.5). The variables for the entire cohort and for each group are summarized in Table 1. There were more males in the case group than in the control group (*p* = 0.049), but no significant differences in grouping was shown based on age. As also shown in Table 1, the QRF muscle area and thickness significantly decreased (*p* ≤ 0.001) and the central tendon thickness significantly increased in the case group when compared to the control group (*p* = 0.047). Moreover, 24.1% of the cases had significantly lower QRF muscle angiogenic activity than the control patients, who had normal vascularization (*p* < 0.001). Similarly, 24.1% of the cases had significantly lower numbers of observed fasciculations compared to 100% of healthy controls (*p* < 0.001). Significantly greater levels of subcutaneous edema and intramuscular fluid (*p* < 0.001) were observed in 88.5% and 69.2% of the cases, respectively, versus 0% for both factors in the control group. Echogenicity was significantly different in the cases when compared to the controls (*p* < 0.001). None of the cases were graded as Category 1, whereas 13.8% had muscle heterogenecity, and thus were graded as Category 2; 51.7% had fat infiltration (Category 3); and 34.5% had muscle necrosis and fasciitis (Category 4).

The clinical characteristics of the cases are shown in Table 2. The patients were acutely ill on admission, as depicted by the severity scores obtained. Specifically, 86% of the patients had multiorgan failure—all of them had respiratory failure, and approximately 70% also had cardiovascular or renal failure. In addition, 80% of the patients were septic at ICU admission, and 40% were administered corticosteroid treatment or neuromuscular blocking agents during their stay. The mean time between ICU admission, performance of the QRF ultrasound study, and clinical suspicion of neuromuscular acquired weakness was 28 days (IQR: 16; 47).

As shown in Table 3, the selected variables in the multivariate logistic regression were muscle area (OR per cm^2^ = 0.071; 95% CI = 0.012–0.410) and central tendon thickness (OR per mm = 1.887; 95% CI = 2.661–13.38092). The muscle wasting score, obtained by means of the logistic regression model, was as follows:

Muscle wasting score=−2.641×(QRF Muscle area)+7.543×(QRF Tendon Thickness)

For this score, the area under the ROC curve was 0.902 (95% CI = 0.817–0.987). The threshold that satisfied the criteria of being closest to the upper left-hand corner was 0.537.

Table 4 shows the estimation of sensitivity and specificity. The predictive values shown correspond to a prevalence of 60.4%, which is the proportion of cases (*n* = 29) versus the total number of individuals studied (*n* = 48).

## 4. Discussion

Sepsis, difficult ventilator liberation, and prolonged mechanical ventilation are among the well-known risk factors associated with neuromuscular acquired weakness [26]. Aggressive nutritional therapy and early exercise should be considered to prevent muscle wasting or to maintain muscle, although they may fail when applied early in critically ill patients [2,3]. However, BMI assessment does not indicate the muscle quality and quantity of wasting in these patients. In contrast, US is a valuable tool that allows the morphological changes of muscles to be assessed in septic patients [27].

On applying the newly designed QRF-US muscle protocol in long-term, ventilated, critically ill patients, we found that QRF muscle area and thickness significantly decreased in the studied cases when compared to the control group (*p* ≤ 0.001) [28,29,30,31]. However, more importantly, the QRF central tendon thickness significantly increased in the cases (*p* = 0.047). This finding suggests that, due to its dense connective tissue nature, QRF central tendons did not suffer atrophy, and thus became sonographically more evident in patients with QRF muscle wasting.

The sonographic importance of QRF is linked to its special anatomic nature. The QRF is the only anatomic bipennate muscle with a unique intramuscular central tendon between the four quadriceps femoris muscles, whose muscle fibers are obliquely inserted at both tendon sites. Furthermore, QRF is the only quadriceps femoris muscle with extrinsic activity, and it contains type IIA, IIX, and IIB fast-twitch fibers, which are thicker and quicker to contract, as well as being the most powerful muscle type with the lowest endurance. Thus, this muscle activates when the body nears maximum exertion [32,33]. In our study, we demonstrated that when QRF muscle wasting is present in critically ill patients with prolonged mechanical ventilation, the QRF central tendon size is considerably increased. Although an association between QRF central tendon injury at the proximal level and a greater absence time in sports participation has already been demonstrated [14], this finding was depicted as a visible and sizeable echogenic linear US image. This is the first time, to our knowledge, that a relevant contribution of the QRF central tendon has been described when diagnosing muscle wasting in critically ill patients.

Nevertheless, skeletal muscle function depends not only on its quantity, but also on its quality, which may be adversely affected. Echo-intensity assessment is the most difficult measurement to make, and needs specific training to achieve reproducibility [34,35]. Changes in US-assessed QRF muscle echogenicity and fascial characteristics have been linked to microscopic, histologically-defined myofiber necrosis and fascial pathology [20]. In this study, non-specified changes in muscle US echogenicity were more common in patients who developed muscle necrosis than in those who did not. The authors conclude that myofiber necrosis and fascial inflammation can be detected non-invasively using US in critically ill patients, and that fasciitis usually precedes or accompanies muscle necrosis [36].

As previously discussed, during the acute phase of a severe catabolic illness, normal muscle is gradually replaced by fat or fibrous tissues, increasing its echogenicity. When a partial fat muscle or a fibrous infiltration is produced, sonographic assessment initially shows it to be heterogeneous, and later on, echogenic, once it is completely infiltrated by fat. Eventually, it is impossible to differentiate the muscle due to fasciitis and myofiber necrosis, which is shown at more advanced stages of wasting [18]. However, in our study, we were able to categorize these morphological changes found in the QRF muscle in four grades, using only US, and biopsies were not required. We observed that the QRF echogenicity was significantly different in the cases when compared to the controls (*p* < 0.001). Specifically, 13.8% of the cases had muscle heterogeneity, 51.7% had fat infiltration, and 34.5% had muscle necrosis and fasciitis, depicting the evolving deep morphological changes that take place in the muscle wasting process. Finally, the healthy controls were all hypoechoic (normal echogenicity).

We also visualized QRF muscle vascularization with color Doppler US, which allowed us to more actively assess blood flow changes in the studied subjects. Significantly lower QRF muscle angiogenic activity was observed in 24.1% of the cases compared with the controls, who had normal vascularization (*p* < 0.001). This reduction in the muscle vascularization of the cases is considered secondary to a lowering of muscle angiogenesis, because of the replacement process of the QRF muscle by fat and connective tissue [37].

Muscle fasciculations represent spontaneous activity in the muscle, and are a sign of increased excitability of impaired motor nerves. US captures the mechanical behavior of the muscle, whereas an electromyogram (EMG) only captures brief changes in the muscle membrane potential [18]. In addition to this, the detection of muscle fasciculations can be visualized with higher sensitivity by US compared to an EMG, as a larger muscle area can be assessed [27]. We applied M-mode US to visualize QRF muscle fasciculations, and observed that 24.1% of the cases had significantly fewer fasciculations compared to 100% of healthy controls (*p* < 0.001). This lessening or absence of detected fasciculations may be explained by muscle-specific myofiber alterations [27].

Muscle is mainly composed of water, representing around 30% of the body water reserve, and may significantly change with dehydration and fluid overload [18]. US can easily detect fluid displacement in critically ill patients by visualizing the presence of intramuscular and interfascial fluid overload and subcutaneous edema. These factors have been described in critical patients with positive fluid balance and with septic shock in the acute phase [11]. Our data corroborated these findings, showing significantly more subcutaneous edema and intramuscular fluid in the cases, compared to their absence in the control group (*p* < 0.001).

Finally, in our study, the variables selected in the multivariate logistic regression analysis were QRF muscle area and the central tendon thickness, as discussed above. Furthermore, through that analysis, we also obtained a “muscle wasting score”, with an area under the ROC curve of 0.902 (95% CI = 0.817–0.987), which we consider to have an excellent predictive value that will help clinicians to diagnose muscle wasting in patients of the type described here.

## 5. Conclusions

A well-designed US protocol allowed us to assess the quantitative and qualitative changes in QRF muscle in severely ill, mechanically-ventilated patients with clinically suspected neuromuscular acquired weakness. Among the US quantitative changes, muscle area and thickness significantly decreased and the QRF central tendon thickness significantly increased in the cases versus the controls. These findings are relevant because they show, for the first time, that the QRF central tendon does not suffer atrophy due to its dense connective tissue nature; this was sonographically more evident in severely catabolically ill patients with QRF muscle wasting. Our protocol also allowed us to search for muscle quality changes, and we found that QRF echogenicity, angiogenic activity, and the presence or absence of muscle fasciculations were significantly different in the cases when compared to the controls, depicting the evolving deep morphological changes reflected in metabolic alterations that were taking place in the sarcopenic process in our patients.

## Figures and Tables

**Table 1:** Characteristics of the cases and controls.

	Controls (*n* = 19)	Cases (*n* = 29)	*p*
Mean age in years (± SD)	59.9 ± 18.4	62.9 ± 15.7	0.543
Age 65 years, *n* (%)	10 (52.6)	14 (48.3)	0.768
Sex, *n* (%)			0.049
Male	5 (26.3)	16 (55.2)	
Female	14 (73.7)	13 (44.8)	
Body mass index, kg/m^2^, mean (±SD)	25.4 ± 3.6	28.3 ± 5.4	0.068
QRF muscle area, cm^2^, median (IQR)	3.6 (3.0; 4.3)	1.0 (0.5; 1.8)	<0.001
QRF tendon thickness, mm, median (IQR)	1.5 (1.4; 1.6)	1.8 (1.3; 2.1)	0.047
QRF muscle thickness, mm, median (IQR)	11.4 (7.5; 13.3)	5.7 (3.5; 8.5)	0.001
Doppler color, *n* (%)	19 (100)	7 (24.1)	<0.001
Fasciculations, *n* (%)	19 (100)	7 (24.1)	<0.001
Subcutaneous edema, *n* (%)	0	23 (88.5)	<0.001
Intramuscular fluid, *n* (%)	0	18 (69.2)	<0.001
Echogenicity, *n* (%)			<0.001
1	19 (100.0)	0	
2	0	4 (13.8)	
3	0	15 (51.7)	
4	0	10 (34.5)	

SD: standard deviation, IQR: interquartile range, QRF: Quadriceps rectus femoris.

**Table 2:** Clinical characteristics of the cases.

	*n* = 29
Apache-II median (IQR)	26.5 (23.8; 29)
SOFA score at ICU admission, median (IQR)	7 (6.2; 7.8)
GCS at ICU admission, median (IQR)	4 (3; 14)
Time between ICU admission and QRF-US in days, median (IQR)	28 (15.5; 48.5)
Multiorgan failure, *n* (%)	25 (86.2)
Respiratory failure, *n* (%)	25 (86.2)
Cardiovascular failure, *n* (%)	21 (72.4)
Renal failure, *n* (%)	20 (69.0)
Hepatic failure, *n* (%)	2 (6.9)
Hematologic failure, *n* (%)	1 (3.5)
Gastrointestinal failure, *n* (%)	1 (3.5)
Septic at ICU admission, *n* (%)	23 (80.1)
Corticosteroids, *n* (%)	11 (37.9)
Neuromuscular blockers, *n* (%)	12 (41.4)

Apache: acute physiology and chronic health evaluation; IQR: interquartile range; SOFA: sequential organ failure assessment; ICU: intensive care unit; GCS: Glasgow Coma Score; QRF–US: quadriceps rectus femoris ultrasonography.

**Table 3:** Multivariate logistic regression.

	Coefficient (SE)	*p*	OR (95% CI)
**(Intercept)**	−4.533 (3.578)	0.205	-
QRF Muscle area, per cm^2^	−2.641 (0.893)	0.003	0.071 (0.012; 0.410)
QRF Tendon Thickness, per mm	7.543 (3.349)	0.024	1887 (2.661; 1,338,092)

QRF: quadriceps rectus femoris.

**Table 4:** Evaluation of the predictive rule for the outcome based on the score (threshold = 0.537).

Parameter	Estimation (%)	95% CI
Sensitivity	90.9	70.8; 98.9
Specificity	89.5	66.9; 98.7
Negative predictive value	89.5	69.2; 97.0
Positive predictive value	90.9	72.8; 97.4

## References

[1] Coelho-Júnior H.J., Rodrigues B., Uchida M., Marzetti E. (2018). Low protein intake is associated with frailty in older adults: A systematic review and meta-analysis of observational studies. Nutrients.

[2] Wischmeyer P.E., Puthucheary Z., San Millán I., Butz D., Grocott M.P.W. (2017). Muscle mass and physical recovery in ICU: Innovations for targeting of nutrition and exercise. Curr. Opin. Crit. Care.

[3] Casaer M.P. (2015). Muscle weakness and nutrition therapy in ICU. Curr. Opin. Nutr. Metab. Care.

[4] Lee S.Y., Ahn S., Kim Y.J., Ji M.J., Kim K.M., Choi S.H., Jang H.C., Lim S. (2018). Comparison between dual-energy X-ray absorptiometry and bioelectrical impedance analyses for accuracy in measuring whole body muscle mass and appendicular skeletal muscle mass. Nutrients.

[5] Schepens T., Verbrugghe W., Dams K., Corthouts B., Parizel P.M., Jorens P.G. (2015). The course of diaphragm atrophy in ventilated patients assessed with ultrasound: A longitudinal cohort study. Crit. Care.

[6] Prado C.M.M., Heymsfield S.B. (2014). Lean tissue imaging: A new era for nutritional assessment and intervention. JPEN J. Parenter. Enter. Nutr..

[7] Weijs P.J., Looijaard W.G., Dekker I.M., Stapel S.N., Girbes A.R., Oudemans-van Straaten H.M., Beishuizen A. (2014). Low skeletal muscle area is a risk factor for mortality in mechanically ventilated critically ill patients. Crit. Care.

[8] Mitsiopoulos N., Baumgartner R.N., Heymsfield S.B., Lyons W., Gallagher D., Ross R. (1998). Cadaver validation of skeletal muscle measurement by magnetic resonance imaging and computerized tomography. J. Appl. Physiol..

[9] Reeves N.D., Maganaris C.N., Narici M.V. (2004). Ultrasonographic assessment of human skeletal muscle size. Eur. J. Appl. Physiol..

[10] Bear D.E., Parry S.M., Puthucheary Z.A. (2018). Can the critically ill patient generate sufficient energy to facilitate exercise in the ICU?. Curr. Opin. Clin. Nutr. Metab..

[11] Fischer A., Spiegl M., Altmann K., Winkler A., Salamon A., Themessl-Huber M., Mouhieddine M., Strasser E.M., Schiferer A., Paternostro-Sluga T. (2016). Muscle mass, strength and functional outcomes in critically ill patients after cardiothoracic surgery: Does neuromuscular electrical stimulation help? The Catastim 2 randomized controlled trial. Crit. Care.

[12] Mourtzakis M., Parry S., Connolly B., Puthucheary Z. (2017). Skeletal muscle ultrasound in critical care: A tool in need of translation. Ann. Am. Thorac. Soc..

[13] Sabatino A., Regolisti G., Bozzoli L., Fani F., Antoniotti R., Maggiore U., Fiaccadori E. (2016). Reliability of bedside ultrasound for measurement of quadriceps muscle thickness in critically ill patients with acute kidney injury. Clin. Nutr..

[14] Balius R., Maestro A., Pedret C., Estruch A., Mota J., Rodríguez L., García P., Mauri E. (2009). Central aponeurosis tears of the rectus femoris: Practical sonographic prognosis. Br. J. Sports Med..

[15] Hermans G., Van den Berghe G. (2015). Clinical review: Intensive care unit acquired weakness. Crit. Care.

[16] Witteveen E., Sommers J., Wieske L., Doorduin J., van Alfen N., Schultz M.J., van Schaik I.N., Horn J., Verhamme C. (2017). Diagnostic accuracy of quantitative neuromuscular ultrasound for the diagnosis of intensive care unit-acquired weakness: A cross-sectional observational study. Ann. Intensive Care.

[17] Hough C.L., Lieu B.K., Caldwell E.S. (2011). Manual muscle strength testing of critically ill patients: Feasibility and interobserver agreement. Crit. Care.

[18] Mayans D., Cartwright M.S., Walker F.O. (2012). Neuromuscular ultrasonography: Quantifying muscle and nerve measurements. Phys. Med. Rehabil. Clin. N. Am..

[19] Tandon P., Low G., Mourtzakis M., Zenith L., Myers R.P., Abraldes J.G., Shaheen A.A., Qamar H., Mansoor N., Carbonneau M. (2016). A Model to Identify Sarcopenia in Patients with Cirrhosis. Clin. Gastroenterol. Hepatol..

[20] Strasse E.M., Draskovits T., Praschak M., Quittan M., Graf A. (2013). Association between ultrasound measurements of muscle thickness, pennation angle, echogenicity and skeletal muscle strength in the elderly. Age.

[21] Rollins K.E., Javanmard-Emamghissi H., Awwad A., Macdonald I.A., Fearon K.C.H., Lobo D.N. (2017). Body composition measurement using computed tomography: Does the phase of the scan matter?. Nutrition.

[22] Aubrey J., Esfandiari N., Baracos V.E., Buteau F.A., Frenette J., Putman C.T., Mazurak V.C. (2014). Measurement of skeletal muscle radiation attenuation and basis of its biological variation. Acta Physiol..

[23] Miljkovic I., Zmuda J.M. (2010). Epidemiology of myosteatosis. Curr. Opin. Clin. Nutr. Metab. Care.

[24] Fujiwara N., Nakagawa H., Kudo Y., Tateishi R., Taguri M., Watadani T., Nakagomi R., Kondo M., Nakatsuka T., Minami T. (2015). Sarcopenia, intramuscular fat deposition, and visceral adiposity independently predict the outcomes of hepatocellular carcinoma. J. Hepatol..

[25] Mohseni-Bandpei M.A., Nakhaee M., Mousavi M.E., Shakourirad A., Safari M.R., Vahab Kashani R. (2014). Application of ultrasound in the assessment of plantar fascia in patients with plantar fasciitis: A systematic review. Ultrasound Med. Biol..

[26] Fan E., Cheek F., Chlan L., Gosselink R., Hart N., Herridge M.S., Hopkins R.O., Hough C.L., Kress J.P., Latronico N. (2014). ATS Committee on ICU-acquired weakness in adults; American thoracic society. An official American thoracic society clinical practice guideline: The diagnosis of intensive care unit-acquired weakness in adults. Am. J. Respir. Crit. Care Med..

[27] Grimm A., Teschner U., Porzelius C., Ludewig K., Zielske J., Witte O.W., Brunkhorst F.M., Axer H. (2013). Muscle ultrasound for early assessment of critical illness neuromyopathy in severe sepsis. Crit. Care.

[28] Gruther W., Benesch T., Zorn C., Paternostro-Sluga T., Quittan M., Fialka-Moser V., Spiss C., Kainberger F., Crevenna R. (2008). Muscle wasting in intensive care patients: Ultrasound observation of the M. quadriceps femoris muscle layer. J. Rehabil. Med..

[29] Tillquist M., Kutsogiannis D.J., Wischmeyer P.E., Kummerlen C., Leung R., Stollery D., Karvellas C.J., Preiser J.C., Bird N., Kozar R. (2014). Bedside ultrasound is a practical and reliable measurement tool for assessing quadriceps muscle layer thickness. J. Parenter. Enteral Nutr..

[30] Puthucheary Z.A., Rawal J., McPhail M., Connolly B., Ratnayake G., Chan P., Hopkinson N.S., Phadke R., Dew T., Sidhu P.S. (2013). Acute skeletal muscle wasting in critical illness. JAMA.

[31] Parry S.M., El-Ansary D., Cartwright M.S., Sarwal A., Berney S., Koopman R., Annoni R., Puthucheary Z., Gordon I.R., Morris P.E. (2015). Ultrasonography in the intensive care setting can be used to detect changes in the quality and quantity of muscle and is related to muscle strength and function. J. Crit. Care.

[32] Wilson J.M., Loenneke J.P., Jo E., Wilson G.J., Zourdos M.C., Kim J.S. (2012). The effects of endurance, strength, and power training on muscle fiber type shifting. J. Strength Cond. Res..

[33] Bierbrauer J., Koch S., Olbricht C., Hamati J., Lodka D., Schneider J., Luther-Schröder A., Kleber C., Faust K., Wiesener S. (2012). Early type II fiber atrophy in intensive care unit patients with nonexcitable muscle membrane. Crit. Care Med..

[34] Ticinesi A., Meschi T., Narici M.V., Lauretani F., Maggio M. (2017). Muscle Ultrasound and Sarcopenia in Older Individuals: A Clinical Perspective. J. Am. Med. Dir. Assoc..

[35] Berger J., Bunout D., Barrera G., de la Maza M.P., Henriquez S., Leiva L., Hirsch S. (2015). Rectus femoris (RF) ultrasound for the assessment of muscle mass in older people. Arch. Gerontol. Geriatr..

[36] Puthucheary Z.A., Phadke R., Rawal J., McPhail M.J., Sidhu P.S., Rowlerson A., Moxham J., Harridge S., Hart N., Montgomery H.E. (2015). Qualitative ultrasound in acute critical illness muscle wasting. Crit. Care Med..

[37] Batt J., Mathur S., Katzberg H.D. (2017). Mechanism of ICU-acquired weakness: Muscle contractility in critical illness. Intensive Care Med..

